# Passive Sampler Technology for Viral Detection in Wastewater-Based Surveillance: Current State and Nanomaterial Opportunities

**DOI:** 10.3390/v15091941

**Published:** 2023-09-16

**Authors:** Alberto Aguayo-Acosta, Mildred G. Jiménez-Rodríguez, Fernando Silva-Lance, Mariel Araceli Oyervides-Muñoz, Arnoldo Armenta-Castro, Orlado de la Rosa, Antonio Ovalle-Carcaño, Elda M. Melchor-Martínez, Zahra Aghalari, Roberto Parra-Saldívar, Juan Eduardo Sosa-Hernández

**Affiliations:** 1 Institute of Advanced Materials for Sustainable Manufacturing, Tecnologico de Monterrey, Monterrey 64849, Mexico; aguayo.alberto@tec.mx (A.A.-A.); mariel.oyervides@tec.mx (M.A.O.-M.); orlando.delarosa@tec.mx (O.d.l.R.); antonio.oc95@tec.mx (A.O.-C.); elda.melchor@tec.mx (E.M.M.-M.); 2 School of Engineering and Sciences, Tecnologico de Monterrey, Monterrey 64849, Mexico; A01631207@tec.mx (M.G.J.-R.); A01704893@tec.mx (F.S.-L.); A01740937@tec.mx (A.A.-C.); 3 Faculty of Public Health, Babol University of Medical Sciences, Babol 47176-47754, Iran; z.aghalari@gmail.com

**Keywords:** waterborne viruses, nanostructured materials, passive samplers, solid surface adsorption, wastewater-based surveillance

## Abstract

Although wastewater-based surveillance (WBS) is an efficient community-wide surveillance tool, its implementation for pathogen surveillance remains limited by ineffective sample treatment procedures, as the complex composition of wastewater often interferes with biomarker recovery. Moreover, current sampling protocols based on grab samples are susceptible to fluctuant biomarker concentrations and may increase operative costs, often rendering such systems inaccessible to communities in low-to-middle-income countries (LMICs). As a response, passive samplers have emerged as a way to make wastewater sampling more efficient and obtain more reliable, consistent data. Therefore, this study aims to review recent developments in passive sampling technologies to provide researchers with the tools to develop novel passive sampling strategies. Although promising advances in the development of nanostructured passive samplers have been reported, optimization remains a significant area of opportunity for researchers in the area, as methods for flexible, robust adsorption and recovery of viral genetic materials would greatly improve the efficacy of WBS systems while making them more accessible for communities worldwide.

## 1. Introduction

Wastewater-based surveillance (WBS) is a novel tool for public health assessment and insurance that consists in the sustained monitoring of pathogenic agents in sewage by tracing the abundance and diversity of relevant biomarkers to aid in epidemiological surveillance and the prevention and containment of outbreaks [1,2]. In most cases, WBS systems require periodic sampling of wastewater treatment plants (WWTP) and the use of polymerase chain reaction (PCR)-based assays to detect the presence of a given pathogen of interest [1]. Several studies have detected the presence of viral pathogens in sewage through WBS, linking them to local outbreaks and environmental contamination [3,4,5,6]. However, WBS systems have also been successfully implemented to track the circulation of different xenobiotics in specific populations, such as pharmaceuticals, recreative drugs, and personal care products, among others [7,8].

A notable example of the successful application of WBS systems for monitoring the development of community-wide infections is the case of the COVID-19 pandemic, caused by the novel severe acute respiratory syndrome coronavirus (SARS-CoV-2) [9,10], where they were used to estimate the distribution and temporality of SARS-CoV-2 infections within the catchment area of WWTPs [11], as early reports documented the detection of SARS-CoV-2 in the urine and stool samples of infected patients, even if nasopharyngeal tests showed negative results, which was later confirmed by several research groups around the world, supporting the feasibility of using WBS for the longitudinal monitoring of viral genetic materials to estimate the incidence and persistence of specific pathogenic viruses in a given population to guide strategic plans toward pandemic preparedness [12,13,14,15,16,17]. WBS has also been used to monitor the incidence and prevalence of other human pathogens in communities, including human norovirus [18], hepatitis A virus (HAV) [19], enteroviruses, and adenoviruses [20], among others.

Nonetheless, effective sample preconcentration and genetic material recovery methods remain one of the greatest areas for improvement in WBS applications, as the reliable detection of viruses in matrices as highly complex as wastewater is crucial for epidemiological surveillance [21,22]. Currently, the most commonly used approaches for sample concentration and virus recovery use membrane filtration and rely mainly on managing different physicochemical interactions between the target molecule and the filter [21]. Two common examples of these membrane-based recovery techniques are virus adsorption–elution (VIRADEL) and crossflow ultrafiltration (CFUF), which are usually used for primary concentration of environmental samples [21,22]. Secondary concentration steps consist in further reducing the sample’s volume so that it is suitable for analyses such as qPCR [18].

Although these recovery techniques have been useful for the detection of viruses in wastewater, fluctuations in biomarker abundance and the complex composition of sewage usually result in low, poorly reproducible recoveries, as different types of molecules commonly suspended or dissolved in wastewater samples are likely to foul the filters, limiting recovery yields and potentially interfering with downstream assays [21,23,24]. In addition, some filters may require preconditioning steps to facilitate adsorption, which is often time-consuming and may affect the integrity of the molecule of interest, hindering the accurate characterization of the sample [21].

Another important factor for the success of WBS is the implementation of appropriate spatial and temporal sampling strategies [1,23]. Most protocols for viral pathogen surveillance use grab samples taken from WWTPs, either by hand or, in some cases, with the use of autosampler devices [13,16]. Typically, plastic or glass containers are filled with wastewater and transported to centralized laboratories where analytical assays are performed [16]. In such cases, results are only representative of the conditions at the moment in which samples were taken, giving rise to the over- or underestimation of the actual concentration of the pathogen of interest [5]. Furthermore, personnel involved in sampling are exposed to waterborne infectious agents present in wastewater (e.g., enteroviruses, pathogenic bacteria), which is a significant health hazard [1,3,4,25,26]. Integrating passive sampling devices into longitudinal pathogen surveillance studies in wastewater, either at WWTPs or in specific sampling points within the sewage system, can improve data reliability as genetic materials accumulate on the device over an extended period (often around 24 h) and can effectively survey both the solid and liquid fractions of the flow, characteristics identified as necessary for robust pathogen surveillance platforms in previous studies [27]. Repeated observations of pathogen circulation and abundance can be integrated into comprehensive statistics databases to draw temporal trends, which are useful for decision makers, while reducing occupational risks by significantly reducing exposure of the involved personnel to waterborne pathogens, although adequate safety measures should still be observed [5,28].

Passive samplers are devices designed to selectively accumulate different types of labile biomarkers in a stable form for their subsequent quantification [27]. As a response to the COVID-19 pandemic, passive sampler devices have been developed and applied to monitor SARS-CoV-2 [28], as well as influenza A (HAV) and B (HBV), human adenovirus (AdV), human norovirus G11 (NoVGII), measles virus (MeV), human fecal markers crAssphage and pepper mild mosaic virus (PMMoV), *Pseudomonas* spp. Phi6 bacteriophage [29], norovirus, and ostreid herpesvirus type 1 in seawater environments [6]. On the other hand, passive sampling devices have been widely used to monitor a wide array of organic and metallic contaminants in fresh water and seawater [5,27]. In particular, membrane-based passive samplers have proven to be suitable to monitor water for prolonged periods of time, increasing sampling efficiency in terms of time, labor, operative costs, and result representativeness when compared to grab samples, as time-weighted average (TWA) concentrations can be calculated [30]. Membrane adsorption is carried out by connecting functional groups on the surface and pore wall of the polymer membranes that allow for the selective adsorption of the target analytes [31], including organic matter, heavy metals, polyphenols, or naphthalenes, among others [32,33,34,35].

Correctly choosing the absorbent material is vital for adequate viral recovery. For instance, bacteriophage MS2, rotavirus, bacteriophage f2, and poliovirus-1 have all been removed from water solutions with the help of different adsorbents [36], and cellulose ester-based polymeric hydrogels and streptavidin-wrapped nanoparticles (NPs) have been used to detect the ORF1ab and N genes of SARS-CoV-2, revealing a 100% analytic sensitivity and specificity [37]. Similar hydrogels were used to detect porcine parvovirus (PPV), a non-enveloped virus, and Sindbis virus, an enveloped virus [38]. Finally, there is evidence of the usage of a nano-TiO_2_ membrane for virus detection in drinking water [39].

This review aims to provide the reader with an overview of the different types of existent passive sampling devices and their implementation in monitoring the circulation of viral genetic material in aqueous matrices, with an emphasis on wastewater. In addition, the most recent developments in membrane-based passive sampling technologies are discussed as well, in order to evaluate their potential implementation in viral pathogen monitoring strategies. Finally, this study seeks to provide the reader with a clear understanding of the process entailed in the design and application of passive samplers to quantify viral loads in wastewater for the prevention of infectious disease outbreaks.

## 2. Types of Passive Sampling Technologies and Their Potential to Monitor Pathogens through WBS

Several types of absorbent materials have been applied for the development of passive samplers for viral pathogen detection in wastewater, including glass beads, Zetapor membranes, nylon-based materials, low-density polyethylene, polyvinylidene difluoride materials, Moore swabs, 75 mm by 75 mm medical gauze swabs, electronegative filter membranes (cellulose nitrate filter and cotton buds) [40], and polyethylene-based plastic devices [41]. While they have been successfully used for the monitoring of several pathogens, including SARS-CoV-2, novel materials with higher absorption capacities and easy recovery of viral genetic materials are still needed for reliable, consistent, and reproducible detection in WBS systems [42].

### 2.1. Nanostructured-Based Passive Sampling Configurations and Their Potential to Monitor Pathogens through WBS

Based on their composition, the membranes used for passive sampling processes can be classified into inorganic and polymeric membranes; while inorganic membranes are preferably used in applications with harsh environmental conditions, polymeric membranes are more common for water treatment applications [43,44]. One of their main advantages is their high adaptability, as pore sizes can be controlled and the properties of the membrane can be easily modified by adjusting casting and crosslinking conditions, monomer molecules, additives, and concentrations [44,45]. On the other hand, some of their main disadvantages include their susceptibility to fouling due to their inherent hydrophobicity, their low resistance to chlorine, and a strong trade-off between permeability and selectivity [45,46]. Nonetheless, advances in nanotechnology have helped overcome such difficulties [45].

Nanoparticles present advantageous structural, thermal, and mechanical properties that are useful for improved membranes [47,48]. Different types of nanocomposites can be developed according to the membrane structure and location of the nanoparticles; in general, their addition to polymeric membranes contributes to the enhancement of their chemical and mechanical properties (e.g., overcoming their propensity to fouling) [49,50]. Nanomaterials usually allow for better recoveries when compared to traditional adsorption materials since they provide a larger surface area and enhanced adsorption capacity. In addition, their structure can be tailored to provide protection to the sampled genetic material. Therefore, the development of nanostructured polymeric membranes that could be used as passive samplers to monitor viral pathogens (e.g., SARS-CoV-2, waterborne viruses) through WBS would be of great benefit to support public health efforts to prevent infectious disease outbreaks in specific communities and could also evolve to become biosensors for stand-alone pathogen detection [50].

### 2.2. Application of Passive Sampler-Based Viral Pathogen Monitoring Systems in WBS

As reported in Table 1, there have been several reports of the application of passive sampler devices for the monitoring of viral pathogens in wastewater samples. In a study by Hayes et al. [51], a COVID-19 sewer cage (COSCa) was constructed as a passive sampler to survey SARS-CoV-2 circulation in wastewater through four different low-cost, easily accessible sorbents: cellulose sponges, 100% cotton gauze, 100% cotton cheesecloth, and electronegative filter membranes. Bench scale experiments and field studies were carried out as follows: first, deionized (DI) water samples were spiked with heat-inactivated SARS-CoV-2 (HI-SCV-2) and left stirring at room temperature to equilibrate; each adsorbent was then placed inside a COSCa and suspended in the sample and continuously stirred for 24 h at room temperature; then, the COSCa samplers were deployed for 24, 48, and 72 h in two sewers at targeted institutional-level sampling sites. Three different eluents were tested (a lysis buffering agent, a Tween^®^20-based buffer, and a 1:1 acetonitrile–water mixture), and RNA extractions were performed using a magnetic-bead-based protocol, while quantification was performed through quantitative reverse transcription PCR (RT-qPCR). In the case of the DI spiked water samples, the electronegative filters resulted in the highest mean RNA concentration, followed by cheesecloth, while the cellulose sponge resulted in the lowest mean RNA concentrations. On the other hand, for the wastewater field samples, the highest RNA concentration was recovered from the cheesecloth, followed by the electronegative filters [51].

In a study by Kevill et al. [29], Tampax Super Compak Tampons and Whatman SG81 Si–cellulose ion exchange papers were recently employed as passive samplers to evaluate the recovery efficiencies of nine wastewater-associated viruses: influenza A and B, SARS-CoV-2, human adenovirus (AdV), human norovirus G11 (NoVGII), measles virus (MeV), and human fecal markers crAssphage and pepper mild mosaic virus (PMMoV), as well as *Pseudomonas* spp. Phi6 bacteriophage. Samples of wastewater and distilled water ${(dH}_{2}O$) were used; each water type was spiked to reach final concentrations between ${1\times 10}^{4}–{1\times 10}^{5}$ genome copies (gc)/mL of each virus. The passive samplers were placed inside 100 mL aliquots of each sample and left at room temperature (20 °C) for 1 h. Four different viral recovery methods were explored: (1) phosphate-buffered saline (PBS) elution into polyethylene glycol (PEG) precipitation, (2) beef extract (BE) elution into PEG precipitation, (3) no elution into PEG precipitation, and (4) direct extraction. In all cases, NucliSens lysis buffer for nucleic acid extraction was used, followed by qPCR analysis. Tampon passive samplers presented improved recovery data over Whatman ion exchange paper, while the extraction methods that presented higher viral recovery yields were: (3) no elution into PEG precipitation and (4) direct extraction. However, results showed that wastewater samples presented lower recoveries than other samples, probably because chemicals and other contaminants present in such matrices can interfere with the adherence of viruses to the passive samplers or even inhibit downstream quantification processes [29].

In a study by Breulmann et al. [41], a long-term passive sampler was developed based on the application of eight 1.5 m long polyethylene plastic strips and deployed in the inflow section of a WWTP. Monitoring was carried out to obtain three 24 h composite samples per week. Viral genetic material was recovered by washing the polyethylene strips with TM buffer (Tris-HCl and magnesium sulphate solution) and mixing with a vortex to recover the supernatant by centrifugation, which was then precipitated using a solution of PEG and NaCl. RNA extraction was carried out with an RNeasy Microbiome Kit, and SARS-CoV-2 was detected through RT-qPCR targeting of the virus’s E gene. The resulting passive sampling protocol, suitable for long-term deployment, reached a limit of detection of 2.53 copies of the viral genome per liter [41].

## 3. Viral Adsorption onto Solid Materials, Kinetic Adsorption, and Mathematical Models to Understand the Viral Adsorption in the Sampler Matrix

The main purpose of passive sampler devices is to provide representative information for a given period while avoiding direct human contact with potentially hazardous wastewater flows, so extensive characterization of the adsorbent–membrane interactions is required for this to be achievable. Common approaches to study molecule adsorption onto solid systems include kinetics and isotherm experiments. Kinetics experiments are performed to determine the overall adsorption rate, as well as to monitor the adsorption process over time under a given set of environmental conditions. For virus adsorption, the most common models to describe adsorption processes are the pseudo-first-order (PFO) and pseudo-second-order (PSO) models (Table 2), which describe adsorption phenomena as a chemisorption process where adsorption is irreversible. While the PFO model describes a contact-dependent adsorption process limited by the diffusion of the pollutant that is adsorbed onto the adsorbent, the PSO model is used to describe an adsorption process limited by the number of active sites on the absorbent. Both models have been widely applied to study the kinetic behavior of the removal of different pollutants in aqueous environments by a broad range of adsorbents [52].

In parallel, adsorption isotherms are used to determine the adsorption capacity and the performance of adsorbents at a constant temperature and can be represented by different models, the Freundlich and Langmuir isotherm models (Table 3) being the ones that have been most commonly applied for wastewater treatment operations [59]. Each model describes the adsorption process under different assumptions; for example, the Langmuir isotherm model assumes that adsorption takes place on a monolayer, which means that once all available active sites of the adsorbent are taken, the adsorption process will end, making the process of adsorption highly dependent on the surface area of the material. Furthermore, this model proposes that the adsorption surface in the adsorbent is distributed homogeneously with identical binding sites across the material and that the only interaction that exists is between the adsorbent molecules and the adsorbent material, while there is no interaction between the adsorbed molecules [60]. On the other hand, the Freundlich isotherm model, which is a modification of the Langmuir model, does take into account multilayer molecule adsorption, which means that interactions exist between the adsorbed molecules, and assumes that the active sites of the adsorbent are distributed heterogeneously; therefore, the diffusion and mass transfer rates between the adsorbent and the adsorbate are limiting parameters for those processes that adjust to this model [60]. These two, along with the linear model, are considered the most common isotherm models to analyze adsorption systems at equilibrium [52].

In summary, kinetics studies aim to analyze and describe the equilibrium conditions of the system, such as the adsorption rate and equilibrium time, while isotherms studies are developed under previously determined equilibrium conditions to evaluate the influence of the adsorbent and adsorbate ratio. The resulting data allow for the determination of the maximum adsorption capacity under the evaluated conditions.

This section aims to provide a clear view of the applications of the most commonly used kinetic and isotherm models upon a broad range of analytes adsorbed onto solid materials, mainly in aqueous matrices. For instance, Zheng et al. [39] used these models to study the adsorption behavior of a nano-TiO_2_ membrane coupling system for virus (phage F2) removal in drinking water. The system reached adsorption equilibrium in 60 min, kinetic experimental results showed that the PSO rate equation fit the data well, and the process followed the Freundlich isotherm [39]. In a similar line, the adsorption of SARS-CoV-2, PMMoV, and CrAssphage virus genetic materials using a passive sampler composed of granular active carbon (GAC) was described by a four-parameter hybrid Freundlich–Langmuir model, as the process was found to be too complex for a two-parameter model [61].

**Figure 1:**
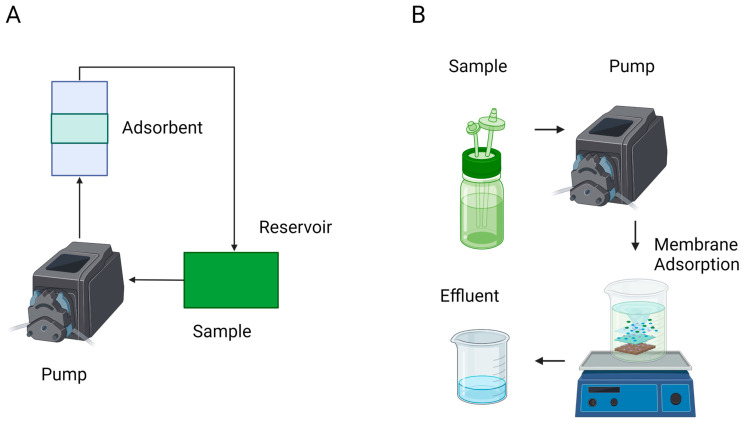
Kinetics experiments in continuous flow system (**A**) and batch reactors (**B**).

During the development of passive samplers, it is important to note that in the case of WBS, the variation of certain parameters, such as pH, temperature, suspended solids, and flow velocity, must be previously studied at a laboratory scale before deployment in the sewage system, to determine if they have a significant impact on the adsorption process; while usage of continuous flow systems would be recommended, as they resemble sewage systems more closely, most studies prefer stirred batch systems, as they are simpler and easier to build while still providing a broad understanding on the capacity of the matrix to adsorb viral materials under specific environments (Figure 1). For reference, while raw wastewater usually has an average pH value of 7.5 [62], variations going from 6.8 to 8.3 have been registered [63]. On the other hand, the minimum flow velocity suggested for pipes in the sewage system is 0.7 m s^−1^, while the recommended range to explore is between 15 °C and 35 °C [64]. Suspended solids’ effects on adsorption have been studied with an initial concentration of 10 mg L^−1^ [65]. It must be noted that, while the results of validation studies using batch systems are mostly consistent with the results of field studies in sewage systems, the capacity of laboratory-scale results to be extrapolated to actual field condition needs to be further studied to obtain better models that could be useful for the design of future devices.

## 4. General Implications in Passive Sampling Design for Viral Monitoring

**Figure 2:**
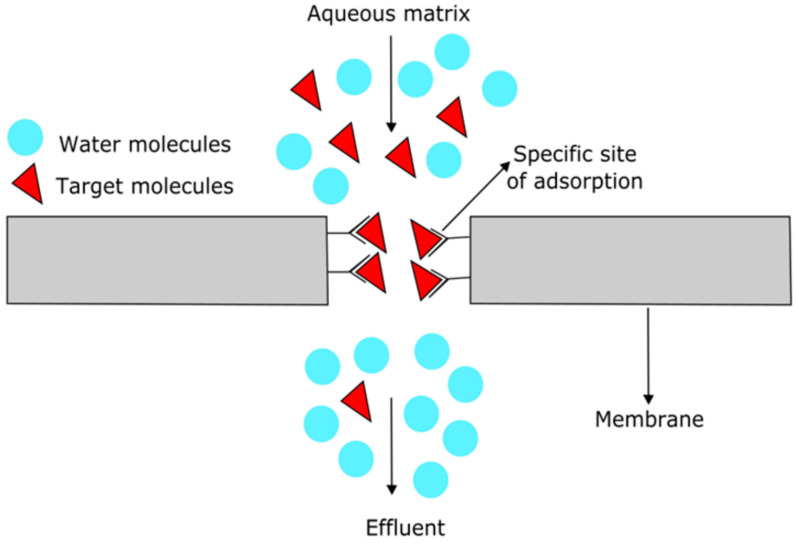
Schematic of membrane adsorption in an aqueous matrix through the interaction of a specific site of adsorption with the target molecule.

Membrane-based passive samplers can be designed by selectively connecting functional groups onto the surface and pores of the polymeric membrane to adsorb the target molecules through the filtration capacities of the membrane, as water flow through the membrane will bind the functional active sites to the target molecules. Figure 2 below shows a schematic of this interaction. However, factors such as temperature, pH, and chemical interactions with the adsorbent material can affect the efficiency of viral genetic material attachment onto the porous solid surface of the membrane, so they must be taken into account during membrane design in order to improve the adsorption rate of viral genetic material onto the sampler [57,66].

Analyzing the potential differences and thermodynamic particularities of viral adsorption onto solid surfaces is important to ensure an optimal design for the passive sampler that allows for rapid and extensive adsorption, as the process is usually driven by electrostatic interactions (i.e., the electrostatic attraction between a virus and an oppositely charged sorbent), which usually results in the adsorption of viral genetic material into the sampler matrix [67]. These interactions are influenced by the pH and ionic strength of the solution [67,68].

Hydrogen bonding is present in terms of viral adsorption onto hydroxyl-containing surfaces and in the presence of aqueous phases; this means that the strength of the bond to the surface would be high in the presence of –O–H···O bonding, especially in environments with a pH higher than 5, where the carboxylic acid functional group present on the surface of the virus is deprotonated [68]. Viruses are typically stable at pH ranges between 5 and 8 and present a net negative charge at neutral pH since they have an isoelectric point below 7, yet viral particles possess a great variety of membrane proteins that can still present positive charges at wider pH ranges, making them more stable [68]. For example, in the case of SARS-CoV-2, this means that the functional groups (−NH_2_, −NH_3_^+^, −COOH, and −COO−) of its surface (S) protein drive adsorption onto solid surfaces through electrostatic interactions between the virion surface’s ionized active species and oppositely charged solids, as well as through hydrogen bonding [68]. Additionally, studies show that exposure to temperatures above 20 °C may decrease the stability of the virus [69]. In any case, while the fractionated particles and genetic material of SARS-CoV-2 may be present, most virions that reach sewage disintegrate when reaching the water effluents [70].

Moreover, while the presence of multiple pathogens in a given water sample increases the need for sensible and specific sampling methods, the introduction of passive sampler devices is a promising strategy for the detection of multiple pathogens, taking into account that multiple detection is comparable to individual virus detection, resulting in flexible, reliable protocols to efficiently detect more than one viral pathogen at a time; such an approach has been used for the surveillance of SARS-CoV-2, influenza A, respiratory syncytial virus, and measles virus through a multiplex RT-PCR assay, reaching a limit of detection of 15 genomic copies per reaction [71].

## 5. Conclusions and Future Perspectives

As shown in the context of the COVID-19 pandemic, there is a need for surveillance and early detection methods to identify, monitor, and limit the spread of infectious disease outbreaks in an effective and efficient manner. While WBS has emerged as a promising set of tools for pandemic preparedness, it still relies heavily on grab sampling that may be hazardous to the involved personnel and may lead to unreliable, inconsistent results. Furthermore, the establishment of adequate WBS infrastructure remains inaccessible for communities in low-to-middle-income countries (LMICs), so novel, cost-effective solutions to monitor viral pathogens to prevent and tackle infectious disease outbreaks are imperative for development in such regions. The introduction of passive samples into WBS methods for viral pathogen surveillance has emerged as a way to improve the early detection of viral waterborne disease outbreaks in vulnerable regions and enhance the detection of diseases in low-incidence settings or in scenarios involving significant proportions of asymptomatic cases while democratizing the access to this technology. Repeated observations of pathogen circulation and abundance can be integrated into comprehensive trends, which are useful for decision makers, while reducing occupational risks associated with grab sampling procedures by significantly reducing personal exposure to waterborne pathogens, although adequate safety measures should be maintained.

After this literature review, it has been concluded that passive samplers represent a promising approach to monitoring pathogenic viruses in wastewater. Several passive samplers were reviewed in this study to evaluate and discuss their technological implications and their capability to assist in the monitoring of viral pathogens using WBS systems. While some of them (e.g., POCIS, DGT) have been commonly used during the last decades, more research regarding technological developments for passive sampling approaches specifically intended to monitor pathogens in aqueous matrices is still needed. In this context, the use of nanostructured materials could be a great area of opportunity due to their enhanced mechanical and chemical characteristics that can be tailored according to the targeted molecule. Appropriate kinetic studies must be conducted during the design process of new passive sampler materials and devices to determine their functional characteristics and ensure optimal functionality. Additionally, further research on viral adsorption in aqueous matrices, especially from wastewater samples, must be conducted to improve the limit of detection that passive sampling devices are able to reach and to ensure robust performance in the context of WBS-based early warning systems.

## Figures and Tables

**Table 1:** Recent examples of passive sampling applications in aqueous matrices.

Adsorbent	Passive Sampling Device	Sorbent(s)	Assay Description /Water Matrix	Sample Extraction Method	Sample Treatment	Analyte Quantification Method/Concentration Calculation	Results	Ref.
SARS-CoV-2 RNA	COVID-19 sewer cage (COSCa)	Cellulose sponge, 100% cotton gauze, 100% cotton cheesecloth, electronegative filter membrane	At laboratory: COSCa were suspended in DI spiked water samples under continuous stirring for 24 h at room temperature. Field study: Devices were deployed for 24, 48, or 72 h in sewer catchments at specific sampling sites.	Elution using a lysis buffering agent, a Tween^®^20-based buffer, and a 1:1 acetonitrile–water mixture.	Magnetic bead-based extraction kit	RT-qPCR RNA concentration (GU per eluate) ≈ sample concentration (GU mL^−1^) × mL of eluate	Electronegative filters and cheesecloth had the highest RNA recovery rate on both spiked DI water and wastewater samples.	[51]
Viral RNA from influenza A and B, SARS-CoV-2, AdV, NoVGII, MeV, PMMoV, crAssphage, and *Pseudomonas* spp. Phi6 bacteriophage	Tampax Super Compak Tampon, 3 cm diameter circular Whatman SG81 Si–cellulose ion exchange paper	Cotton-based tampon, Si–cellulose ion exchange paper	Distilled water and wastewater samples were spiked at concentrations of ~10^4^–10^5^ genome copies/mL for each virus. Unspiked wastewater samples were used as baseline. Samplers were placed into 100 mL aliquots and left at 20 °C for 1 h.	(1) PBS elution into PEG precipitation, (2) BE extract into PEG precipitation, (3) no elution in PEG precipitation, and (4) direct extraction.	NucliSens extraction reagents	qPCR	Tampon passive samplers showed improved recovery over Whatman paper in wastewater samples. No elution and direct extraction resulted in improved viral recoveries. Enveloped viruses showed lower recoveries.	[29]
SARS-CoV-2 RNA	Polyethylene plastic-based passive sampler	Polyethylene	Samplers were deployed for 24 h in specific sampling sites along the sewage system.	TM buffer (50 mM Tris-HCl and 10 mM magnesium sulphate) and precipitation with a solution of PEG and NaCl.	RNeasy Microbiome Kit	RT-qPCR	The sampler had an LOD of 20,000 GC/L in district and whole-city wastewater samples.	[41]

**Abbreviations: AdV**, human adenovirus; **BE**, beef extract; **MeV**, measles virus; **NaCl**, sodium chloride; **NoVGII**, norovirus GII; **PBS**, phosphate-buffered saline; **PEG**, polyethylene glycol; **PMMoV**, pepper mild mottle virus; **RT-qPCR**, retro-transcriptase quantitative polymerase chain reaction; **TM Buffer**, Tris-magnesium buffer; **qPCR**, quantitative polymerase chain reaction.

**Table 2:** Most common models for adsorption kinetics: PFO and PSO.

Model		Constants		Experimental Values	Matrix and Used Material	Ref.
Pseudo-First-Order (PFO) Empirical Model	$\frac{{dq}_{t}}{dt}=k_{1}(q_{e}-q_{t})$	$k_{1}[\frac{1}{h}]$	How fast equilibrium is achieved.	0.032	F2 bacteriophage	[39]
				0.118	Chitosan, wastewater	[53]
				0.823	Chitosan, water	[54]
				3.839	Chitosan	[55]
				6.678	Wastewater, commercial activated carbon (CAC)	[56]
				0.019	Chitosan/cellulose beads	[57]
		$q_{e}[\frac{mg}{g}]$	Equilibrium adsorption capacity.	59.87	Chitosan, wastewater	[53]
				125.20	Chitosan, water	[54]
				63.25 (mg/L)	Chitosan	[55]
				104.20	Wastewater, commercial activated carbon (CAC)	[56]
				45.81	Chitosan/cellulose beads	[58]
Pseudo-Second-Order (PSO) Empirical Model	$\frac{{dq}_{t}}{dt}=k_{2}{\left(q_{e}-q_{t}\right)}^{2}$	$k_{2}[\frac{g}{mg*h}]$	Rate of adsorption equilibrium.	1.85 × 10^−10^	F2 bacteriophage	[39]
				0.0012	Chitosan, wastewater	[53]
				0.0042	Chitosan, water	[54]
				0.0030	Chitosan	[55]
				0.1550	Wastewater, commercial activated carbon (CAC)	[56]
				9.6 × 10^−4^	Chitosan/cellulose beads	[58]
		$q_{e\;}[\frac{mg}{g}]$	Equilibrium adsorption capacity.	1.62 × 10^−9^	F2 bacteriophage	[39]
				61.74	Chitosan, wastewater	[53]
				128.40	Chitosan, water	[54]
				64.14	Chitosan	[55]
				92.50	Wastewater, commercial activated carbon (CAC)	[56]
				192.16	Chitosan/cellulose beads	[58]

**Table 3:** Most common isotherm models: linear, Freundlich, and Langmuir.

Model		Constants		Values in Experiments	Keywords	Ref.
Linear model Empirical isotherm	$q_{e}=KC_{e}$	$C_{e}[\frac{mg}{L}]$	Adsorbate concentration in the medium at equilibrium.	- No values specified, but it was applied in phages adsorption in wastewater as the matrix.	Wastewater, phages	[57]
		$K[\frac{L}{g}]$	Partition coefficient			
			$K=q_{m}\frac{k_{a}}{k_{d}}$ $k_{a}=adsorption\;rate$ $k_{d}=desorption\;rate$			
			$q_{m}=$ maximum adsorption when there is a complete coverage of the surface.			
Freundlich isotherm Empirical isotherm	$q_{e}=K_{F}{C_{e}}^{1/n}$	$K_{F}[\frac{L^{\frac{1}{n}}mg^{1-\frac{1}{n}}}{g}]$	Constant.	27.40	Whole viral particles	[59]
				45.97	Chitosan, wastewater	[53]
				35.72	Chitosan	[55]
				31.80	Wastewater, commercial activated carbon (CAC)	[56]
				2.56	Chitosan/cellulose beads	[58]
		n	Constant.	1.24	F2 bacteriophage	[39]
				3.77	Chitosan, wastewater	[53]
				5.61	Chitosan	[55]
				2.52	Wastewater, activated carbon (CAC)	[56]
				1.49	Chitosan/cellulose beads	[58]
Langmuir model Chemical adsorption model	$q_{e}={(q}_{m}K_{L}C_{e})/(1+K_{L}C_{e})$	$q_{m}[\frac{mg}{g}]$	Maximum adsorption capacity.	121.95	Chitosan, wastewater	[53]
				61.92	Chitosan	[55]
				136.00	Wastewater, commercial activated carbon (CAC)	[56]
				114.47	Chitosan/cellulose beads	[58]
		$K_{L}[\frac{L}{mg}]$	Ratio of adsorption and desorption rate.	0.656	Chitosan, wastewater	[53]
				2.610	Chitosan	[55]
				0.783	Wastewater, commercial activated carbon (CAC)	[56]
				1.297	Chitosan/cellulose beads	[58]
		$C_{e}[\frac{mg}{L}]$	Adsorbate concentration in the medium at equilibrium.	Experimental data	Chitosan, wastewater	[53]

## Data Availability

Not applicable.
